# Annular pancreas concurrent with pancreaticobiliary maljunction presented with symptoms until adult age: case report with comparative data on pediatric cases

**DOI:** 10.1186/1471-230X-13-153

**Published:** 2013-10-25

**Authors:** Long Cheng, Fuzhou Tian, Tiejun Zhao, Yong Pang, Zhulin Luo, Jiandong Ren

**Affiliations:** 1 Department of General Surgery, General Hospital of Chengdu Military Command, Chengdu, Sichuan Province, People’s Republic of China; 2 Dujiangyan aviation medical evaluating and training center of PLA Air Force, Chengdu, Sichuan Province, People’s Republic of China

**Keywords:** Annular pancreas, Pancreaticobiliary maljunction, Pancreatitis

## Abstract

**Background:**

Annular pancreas (AP) concurrent with pancreaticobiliary maljunction (PBMJ), an unusual coexisted congenital anomaly, often presented symptoms and subjected surgical treatment at the early age of life. We reported the first adult case of concurrent AP with PBMJ presented with symptoms until his twenties, and performed a literature review to analyze the clinicopathological features of such cases comparing with its pediatric counterpart.

**Case presentation:**

The main clinical features of this case were abdominal pain and increased levels of plasma amylase as well as liver function test. A complete type of annular pancreas with duodenal stenosis was found, and dilated common bile duct with high confluence of pancreaticobiliary ducts was also observed. Meanwhile, extremely high levels of bile amylase were detected both in common bile duct and gallbladder. The patient received duodenojejunostomy (side-to-side anastomosis) as well as choledochojejunostomy (Roux-en-Y anastomosis), adnd was discharged in a good condition.

**Conclusion:**

AP concurrent with PBMJ usually presents as duodenal obstruction in infancy, while manifests as pancreatitis in adulthood. Careful long-term follow-up is required for children with AP considering its association with PBMJ which would induce various intractable pathologic conditions in the biliary tract and pancreas.

## Background

Annular pancreas (AP) is a rare congenital anomaly that is frequently associated with duodenal atresia (DA) or duodenal stenosis (DS) [1]. Pancreaticobiliary maljunction (PBMJ) is another congenital anomaly defined as an anatomical maljunction of the pancreatic duct and the biliary duct outside of the duodenal wall beyond the influence of the sphincter of Oddi, usually forming a markedly long common channel [2]. PBMJ and AP are embryologically closely related entities [3]; however, there have been only a limited number of case reports. To the best of our knowledge, only 11 pediatric cases have been documented in detail, but no adult case has been reported previously in English literature [4-13]. The authors report the first adult case with a literature review to analyze the clinicopathological features of such cases, and emphasize that the unusual coexistence of the two anomalies should be pay much attention in the diagnosis and treatment of recurrent pancreatitis and in the prevention of cancers originating from bile duct and gallbladder.

## Case presentation

The patient, a 26-year-old man, presented to our department with abdominal pain. He was born at 40 weeks’ gestational age with a birth weight of 3,010 g. Since his 3 years of age, the patient presented with relatively greater appetite compared with corresponding age cohorts and wiggly epigastric mass which would disappear two hours after meal. He remained free from vomiting and acute abdomen until the first episode of abdominal pain at the age of 23 years. Since then, he underwent totally 3 episodes of acute abdomen, and was diagnosed with acute pancreatitis.

**Figure 1:**
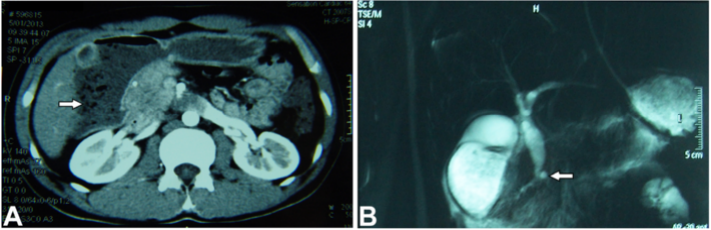
**CT image and MRCP appearance.** Computerized tomography scan showed the dilated descending part of duodenum (arrow) with stenosis at the distal end of dilated duodenum **(A)**. Magnetic resonance cholangiopancreatography revealed fusiform dilatation of the common bile duct with high confluence of pancreaticobiliary ducts (arrow) with a common channel measured 24 mm in length **(B)**.

**Figure 2:**
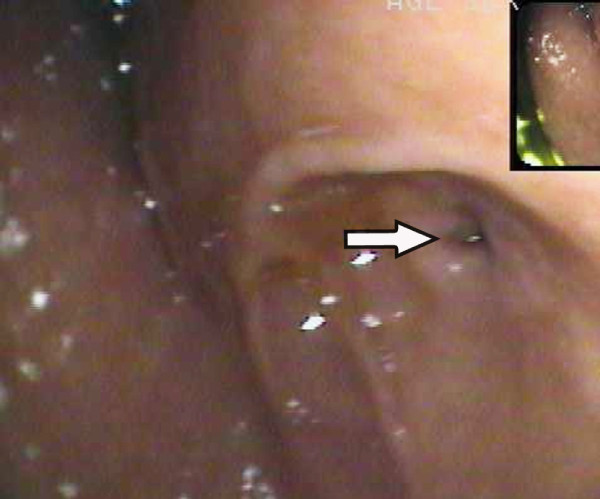
**Endoscopic appearance.** Endoscopy showed an enlarged pylorus and excessively ectatic duodenal cavity. Endoscopic retrograde cholangiopancreatography (ERCP) was not successful in confirming the pancreaticobiliary maljunction due to duodenal stenosis (arrow).

On presentation, physical examination revealed tender epigastrium without palpable mass and jaundice. Laboratory data showed abnormal liver and pancreatic function tests with elevated serum levels of aspartate aminotransferase (85 U/L; normal range, 0–40 U/L), alanine aminotransferase (72 U/L; normal range, 0–40 U/L), total bilirubin (32.7 μmol/L; normal range, 0–19.5 μmol/L), amylase (541 U/L; normal range, 25–125 U/L) and lipase (279 U/L; normal range*,* <190 U/L). Abdominal sonography revealed a dilated common bile duct. Computerized tomography additionally showed the dilation of proximal duodenum with stenosis at the distal end of descending duodenum. Magnetic resonance imaging and cholangiopancreatography revealed fusiform dilatation of the common bile duct with high confluence of pancreaticobiliary ducts with a common channel measured 24 mm in length (Figure 1A, B). Endoscopy showed an enlarged pylorus and excessively ectatic duodenal cavity, but endoscopic retrograde cholangiopancreatography was not successful due to duodenal stenosis at the descending part (Figure 2). A tentative diagnosis of acute pancreatitis associated with pancreaticobiliary maljunction and annular pancreas was made.

**Figure 3:**
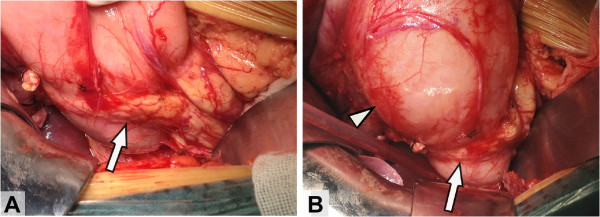
**Intraoperative appearance.** During laparotomy, a complete ring of pancreatic tissue (arrows) surrounded the descending duodenum **(A)**. Gas charging demonstrated duodenal stenosis with dilatation of the stomach and duodenal bulb (triangle) **(B)**.

During laparotomy, a complete type of annular pancreas and a fusiform-type dilatation of common bile duct were confirmed. Air charging demonstrated an enlarged pylorus and excessively ectatic duodenal bulb with duodenal stenosis at the distal end of descending part (Figure 3). Pancreatic amylase was at extremely high levels in the bile within the common bile duct and gallbladder sampled immediately after laparotomy (5592.8 U/L and 85694.0 U/L, respectively). Transection of the dilated common bile duct and choledochojejunostomy were performed. Patient also underwent duodenojejunostomy for correction of duodenal stenosis associated with annular pancreas.

The postoperative course was uneventful. The patient was discharged on the 21th postoperative day on full oral feeding and without abdominal pain. The patient has been free from abdominal pain with normal serum amylase levels in the follow-up period of 12 months postoperatively.

### Discussion

There are three types of pancreatic fusion anomalies: annular pancreas, pancreas divisum and portal annular pancreas [14]. While portal annular pancreas is the rarest and mostly asymptomatic, annular pancreas (AP), with an incidence ranging from 0.005% to 0.015%, usually presents as duodenal obstruction in infancy, [15-17]. However, some patients with AP remain asymptomatic until into adulthood, when the disorder manifests as pancreatitis as shown in our patient. From the data on pediatric cases, it was indicated that pancreatitis in the setting of AP may be associated with concomitant pancreaticobiliary malformations including pancreas divisum, PBMJ, or duodenal diverticulum [18,19], which might induce to insufficient drainage of pancreatic secretions and bile. In the present case, biliopancreatic reflux associated with PBMJ was probably the cause of the development of recurrent pancreatitis.

Until now, there were totally 12 cases of concurrent AP with PBMJ, of which the 11 pediatric cases were described previously, Table 1. Data from these reports showed that most of these patients with AP as well as PBMJ have subjected both duodenoduodenostomy and flow-diversion surgery simultaneously or metachronously. However, prophylactic flow-diversion surgery remains controversial. The recent case reported by Komuro had just received duodenoduodenostomy for AP while prophylactic flow-diversion surgery had not been performed for concomitant PBMJ with bile duct dilatation. According to our experiences, the extremely high levels of pancreatic enzymes in common bile duct or gallbladder should be an important indication for flow-diversion surgery.

**Table 1:** Reported cases of annular pancreas accompanied with pancreaticobiliary maljunction

**Case**	**Author**	**Year**	**Sex**	**Age at duodenal**	**Duodenal Lesion**	**Age at biliary**	**Bile duct lesion associated**
				**surgery**	**associated with AP**	**surgery**	**with PBMJ**
**1**	Komura [5]	1991	F	2 years	DS	2 years	Not dilated
**2**	Okada [4]	1993	M	2 days	DA	12 years	If, IIf
**3**	Okada [4]	1993	F	7 days	DA	3years	If, IIf
**4**	Nakamura [6]	1993	F	3 days	DS	5 years	If
**5**	Komuro [7]	2000	M	11 months	DS	13 months	Intrapacreatic cyst
**6**	Sugimoto [8]	2002	F	0 day	DA	2 years	If, IIf
**7**	Oowari [13]	2003	F	1 day	DA	8 years	If
**8**	Shih [14]	2005	F	3 days	DA	7 years	If, IIf
**9**	Iwai [9]	2009	F	1 day	DA	4 years	If, IIf
**10**	Okuyama[12]	2010	M	neonatal	DA	3 years	If
**11**	Komuro [11]	2012	M	3 years	DS	NOT	Not dilated
**12**	Present case	2013	M	26years	DS	26years	If

M = male; F = Female; DA = duodenal atresia; DS duodenal stenosis, If fusiform dilatation of the common bile duct, IIf fusiform dilatation of the intrahepatic bile duct; NOT = not operated; PBMJ = pancreaticobiliary maljunction.

PBMJ can be divided into PBMJ with biliary dilatation and PBMJ without biliary dilatation [20]. Out of the 12 reviewed cases, 10 cases (83.3%) were found with dilated common bile duct. All seven AP associated DA cases showed fusiform dilatation of the common bile duct at 2–12 years of age. However, no choledochal cyst or marked dilation was discovered soon after birth when they underwent initial surgery for the duodenal obstruction. Thus, it should be suggested that although it is often called “congenital” bile duct dilatation, the bile ducts probably undergo a gradual development of dilatation caused by PBMJ after birth.

The diagnosis of PBMJ in patients with AP is often delayed and even missed, not only because of the gradual and variable development of bile duct dilatation, but also because of lacking accurate diagnostic maneuver for PBMJ [21]. Thus, although combination of AP and PBMJ is a rare condition, careful follow-up is required for the patients with AP, taking into account that PBMJ might present clinical symptoms after several years following definitive surgery for AP. Delayed or missed diagnosis of concurrent PBMJ might sometimes cause serious disease or even death. PBMJ is commonly associated with carcinoma of the bile duct and gallbladder [22,23]. Meanwhile, bile may also reflux into the pancreatic duct via PBMJ in some conditions, such as with cholangitis or bile stasis in the biliary tract. Refluxed bile may activate pancreatic enzymes, and may thus cause recurrent acute pancreatitis and subsequent chronic pancreatitis, which is related to pancreatic carcinoma [24]. Thus, it is very important for AP patients to receive MRCP and/or ERCP to confirm the coexistence of PBMJ, and long-term careful follow-up make much of senses to the prevention and treatment for the recurrent pancreatitis of such patients.

## Conclusions

AP concurrent with PBMJ usually presents as duodenal obstruction in infancy, while manifests as pancreatitis in adulthood. Although prophylactic flow-diversion surgery remains controversial, careful long-term follow-up is required for patients with AP associated DA or DS considering the association of concurrent PBMJ with various intractable pathologic conditions in the biliary tract and pancreas.

## Consent

Written informed consent was obtained from the patient for publication of this Case report and any accompanying images. A copy of the written consent is available for review by the Editor of this journal.

## Abbreviations

AP: Annular pancreas; PBMJ: Concurrent with pancreaticobiliary maljunction; DA: Duodenal atresia; DS: Duodenal stenosis.

## Competing interests

The authors declare that they have no competing interests.

## Authors’ contributions

LC contributed to the design of the study and direction of its implementation. FZT conceived and designed the experiments and supervision of the field activities. LC and TJZ carried out the prepared the Materials of patient and prepared the literature review as well as the Discussion sections of the text. JDR and ZLL conducted the data analysis. All authors read and approved the final version of the manuscript.

## Pre-publication history

The pre-publication history for this paper can be accessed here:

http://www.biomedcentral.com/1471-230X/13/153/prepub
